# Supplementary figures and images for: Real-time structural motif searching in proteins using an inverted index strategy
Source: PLoS Comput Biol. 2020 Dec 7;16(12):e1008502. doi: 10.1371/journal.pcbi.1008502 (PMC7746303; doi:10.1371/journal.pcbi.1008502)

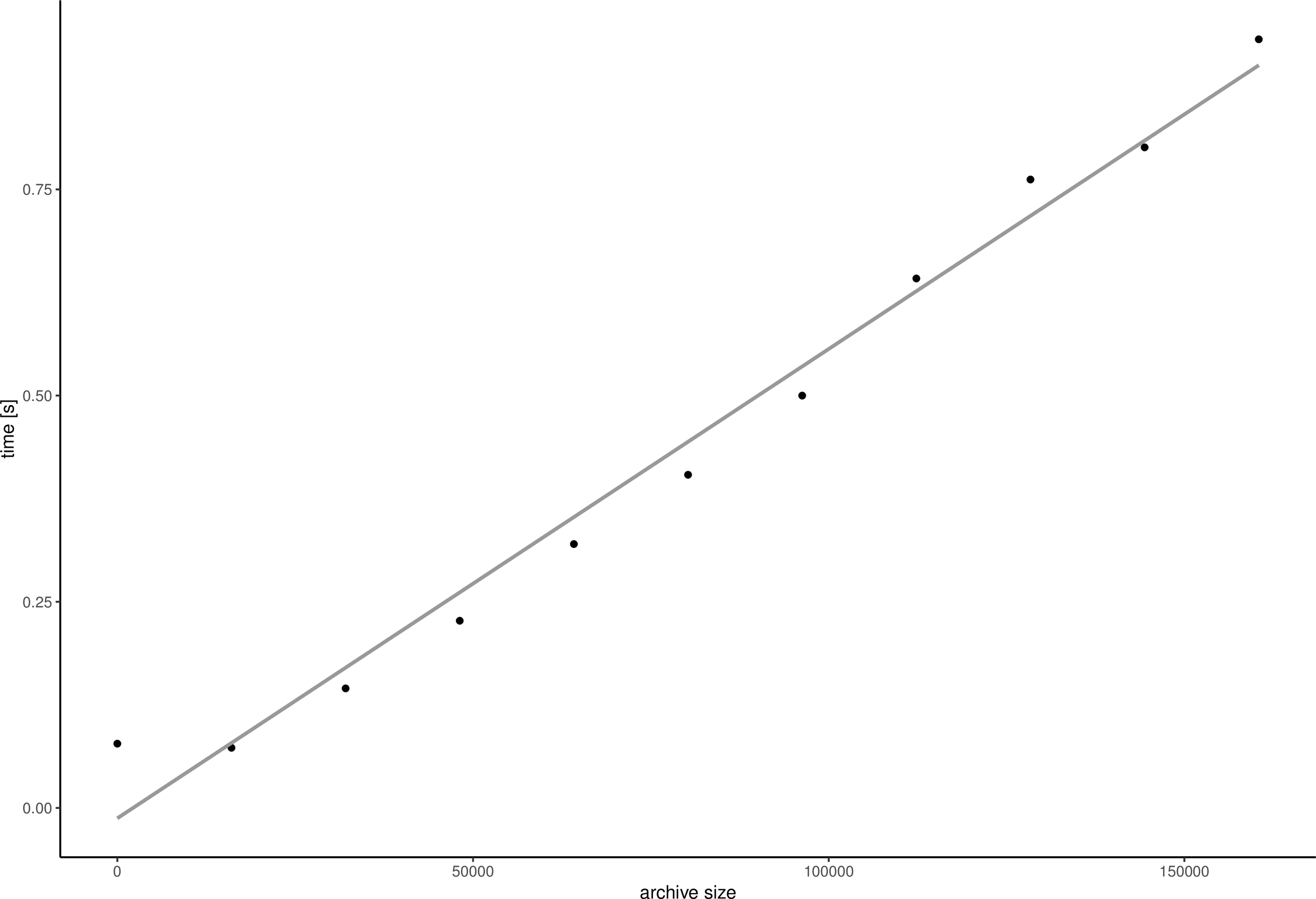

Supplement: S1 Fig — Archive was sampled. Runtime for the catalytic triad query increases linearly with archive size. (TIF) [file pcbi.1008502.s001.tif]

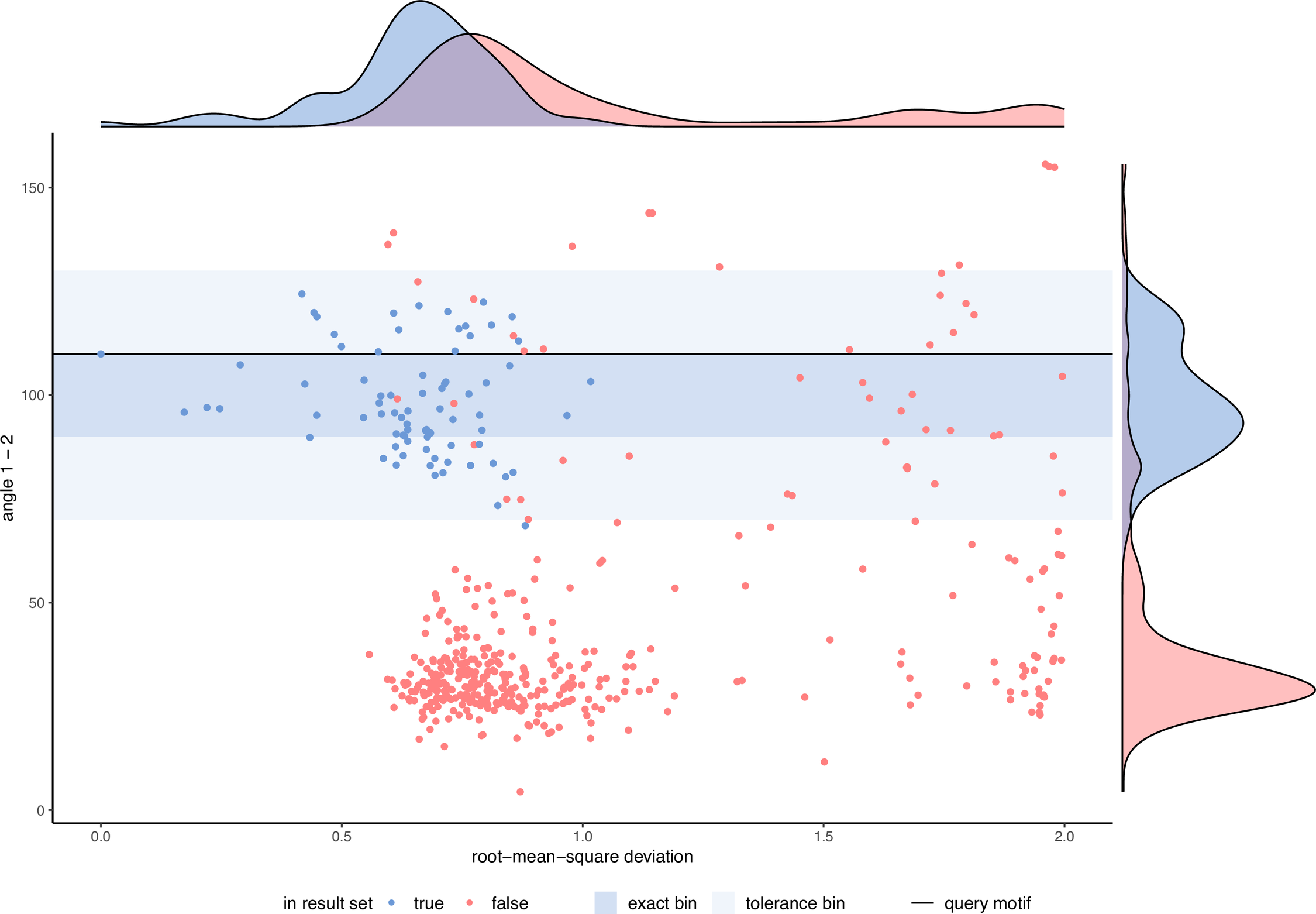

Supplement: S2 Fig — A common motif definition, that encompasses four residues, led to an unacceptable number of false negatives. Cysteine F-212 is structurally variable and most hits were not detected when the angle θ between residues 1 and 2 was below 70°. (TIF) [file pcbi.1008502.s002.tif]

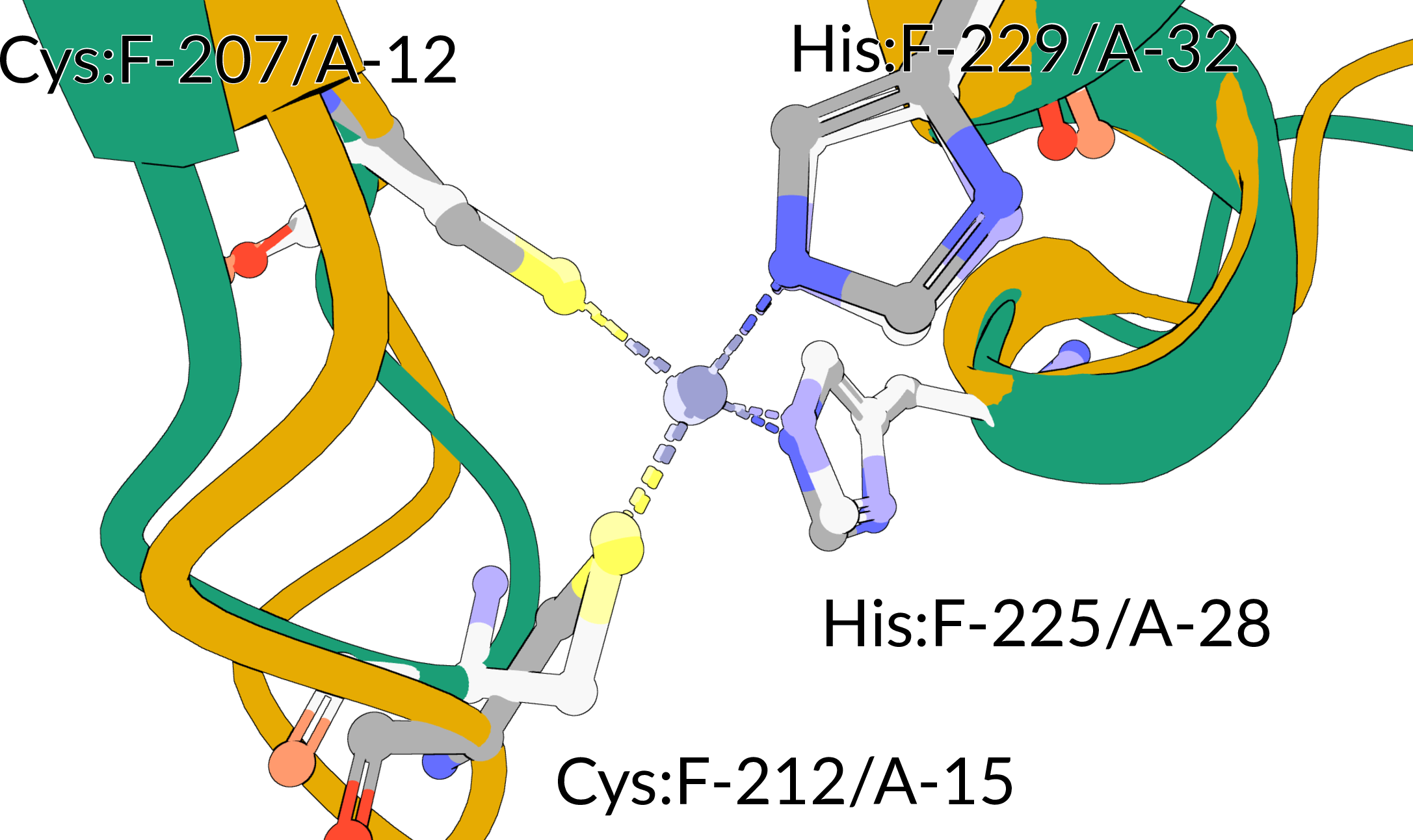

Supplement: S3 Fig — The structurally flexible cysteine at A-15 in PDB ID 2elv (colored in green, motif in light grey) causes this hit to be a false negative. The vector between alpha and beta carbon is orthogonal to that of the query motif. (TIF) [file pcbi.1008502.s003.tif]
