# Supplementary material for: Real-time structural motif searching in proteins using an inverted index strategy
Source: PLoS Comput Biol. 2020 Dec 7;16(12):e1008502. doi: 10.1371/journal.pcbi.1008502 (PMC7746303; doi:10.1371/journal.pcbi.1008502)
Supplement: Attachment — Submitted filename: rebuttal.pdf [file pcbi.1008502.s007.pdf]

Reviewer #1:

Remark 1: *Include the time taken to generate the inverted index, it will presumably need regular updating.*

Response: Updates of the inverted index are an interesting topic and, indeed, integration at RCSB PDB includes a weekly update of the inverted index.

We added the paragraph below to the Results section, providing, anecdotally, wall clock times of a full load and an incremental load (that can readily be performed each week):

- "Individual queries can be processed quickly because the majority of the required computations were performed once during the creation of the inverted index. A full load of 169,117 structures (PDB archive snapshot on 9/25/20) took 3 days and 11 hours. Furthermore, our implementation supports incremental load operations. An incremental load adds the set of PDB identifiers that were deposited since the last update of the inverted index. One week later (on 10/2/20), the incremental load processed 319 structures in 2 hours and 46 minutes. Following the update, all 169,436 were available in the inverted index."

Remark 2: *Github page with software is not currently available*

Response: We will make the GitHub repository public before publication date. Please follow this link to get early access to the code if interested:
https://drive.google.com/file/d/19zp-gEct0sFImS6-ZDT2kmPw6dmf19sw/view?usp=sharing

Remark 3: *More detail on the how the false negative rate was calculated and how common it is to get a high false negative rate as seen with zinc finger motif*

Response: All 3 reviewers indicated that the terminology of "false negative rate" in the context of this manuscript is not as clear as we had hoped. Consequently, we revised the manuscript and:

- Added the following paragraph to the start of the Results section: "We assessed the false negative rate by comparison to an established, exhaustive search strategy represented by Fit3D [Kaiser, 2015], a method based on rigid alignments that scores hits by R.M.S.D. values. We filtered the Fit3D result list for R.M.S.D. values <1 Å to identify true positive hits that our method should report in any case (these hits may not be biologically functional, but should be regarded as promising candidates meriting closer inspection). Our method also finds additional hits because the Fit3D web server operates on a redundancy filtered version of the PDB archive."
- Extended the last subsection of the Methods section by a more detailed description of what "false negatives" means in the context of our manuscript and also addressed the question about the "false positive rate" by the following addition: "[...] overall false negative rate (given as the number of hits reported by Fit3D but missing in result set of our method, divided by the total number of Fit3D hits below the 1 Å R.M.S.D. threshold). We do not discuss false positives (*i.e.*, hits found by our method but not by Fit3D) as they are merely the result of the mandatory redundancy filtering by Fit3D or recent additions to the PDB archive."

We included the case study on the His2/Cys2 zinc fingers to demonstrate the influence of the query motif definition. It is the single example of this that we encountered during development. We cannot provide any empirical data on this issue. However, we would like to point to the first question raised by Reviewer #3 who inquired about possible ways of optimizing motif definitions. As part of the revision, we demonstrate one possible way of refining motif definitions. In future work, we hope to address this issue more thoroughly, *e.g.*, by adding annotations of well-known structural motifs to the RCSB PDB website.

Remark 4: *Include the time taken to generate the inverted index, it will presumably need regular updating.*

See response to Remark 1.

Remark 5: *Grammatical/punctuation etc errors:*

o Management of structure data paragraph: "We this issue by a database"

o Structure of the Inverted Index paragraph: "multiple residue pairs. cases, the inverted indexing strategy" "recent PDB deposited depositions", "6,814,159,549 residues pairs"

Response: Thank you for pointing out these errors, we apologize for missing them during final stages of editing.

We updated the manuscript accordingly:

- Changed to "We addressed this by a database"
- Changed to "multiple residue pairs. In these cases, the inverted indexing strategy"
- Changed to "recent PDB depositions"
- Changed to "6,814,159,549 residue pairs"

Reviewer #2:

Remark 1: *First of all, the only dataset that that manuscript is using to assess the performance of the method is the output of another method, Fit3D. Fit3D is an exhaustive method, but being exhaustive is not being perfect. Every method has its own parameters and performances, therefore I think this point is weak and the work should be strengthened by comparing the results with data extracted from biological databases (PROSITE, with its structural appendix, and other DBs).*

Response: We concur that Fit3D (or any other purely computational method) is not the perfect foundation to evaluate our method. Low geometric dissimilarity (as quantified by R.M.S.D. values), is not necessarily accompanied by biological function or relevance. Vice versa, alignments that result in high R.M.S.D. values are no criteria to rule out biological function. Fit3D tries to mitigate this issue by assigning a statistical probability to hits (similar to the E-value in BLAST runs, see *e.g.* https://doi.org/10.1109/BIBMW.2008.4686202 or https://doi.org/10.1016/S0022-2836(03)00045-7). These statistical models can be considered as helpful indicators but they may be unreliable or not applicable if the result set is too small or exhibits little diversity.

In general, ground truth is difficult to find for the functional annotation of protein structures, especially if this annotation should also identify a set of residues relevant for function. One possibility is the Catalytic Site Atlas (https://doi.org/10.1093/nar/gkx1012). However, only the reference structure is annotated manually and, from there, the function of other structures is inferred by homology. Another possibility would be to use EC annotations. However, again this is not perfect as functionalities such as the given "serine protease" example may be realized by a set of EC numbers (in that case primarily EC 3.4.21.1 and other numbers in EC 3.4.21.X, but this annotation is less clear cut for other structural motifs). The information provided by PROSITE is derived from sequence analysis and may miss more complex motifs that cannot be described adequately by sequence motifs.

To address your concern, we obtained identifier lists for the serine protease example of PDB ID 4cha from CSA, EC, and PROSITE and computed the false negative rate. These results are now presented in the manuscript and resulted in the following changes:

- Added an extra paragraph to the results of the serine protease example: "Additionally, we investigated how well our inverted index method coincides with functional annotation resources ([S3_Table]). Therefore, we collected PDB structures that share an EC number (3.4.21.1), an entry in the Catalytic Site Atlas (M-CSA ID 387, [Ribeiro, 2017]), or a PROSITE pattern (PS50240, [Sigrist, 2012]) with PDB ID 4cha from which the query motif was extracted. For all resources, >90% of hits are found with default parameters. Higher tolerance values result in complete coverage of EC 3.4.21.1 but do not result in substantially higher coverage of M-CSA ID 387 or PS50240. The functional annotations considered are based on homology or sequence patterns and include some occurrences that may not be functionally relevant. For example, the structure of PDB

ID 1a7s aligns well (R.M.S.D.=0.717 Å) but the active site in question exhibits 2 amino acid substitutions. Analogously, the active site of PDB ID 1bio contains a covalently bound inhibitor that may cause an atypical conformation of His:A-57 [Jing, 1998]. Sequence-based methods are orthogonal to structure-based ones, thus, it is advantageous to use multiple resources to screen for protein function [Kirshner, 2013]."

- Added supplemental table S3_Table that contains the new data related to the serine protease example

Remark 2: *The Authors report the number of false negatives that the searches give, never reporting also the false positives. I think that a fair evaluation of this work should contain also MCC or other standard more comprehensive parameters.*

Response: The Fit3D algorithm determines if a certain arrangement represents the query motif based on a rigid structure alignment. Therefore, we considered all reported hits to be valid and wanted to reproduce the result set of Fit3D completely (or miss as few hits as possible). We considered all hits by Fit3D below the R.M.S.D. threshold as "true positives". Furthermore, we did not regard any hits reported by our method as "false positives" if they were not reported by Fit3D. Rather this is the consequence of the mandatory sequence-based redundancy filtering implemented by Fit3D or the result of newly deposited PDB structures not evaluated by the Fit3D web server.

The initial version of our manuscript did not clearly communicate what we mean by "false negative rate" and did not give a reason why we never reported a "false positive rate". We, therefore, revised the manuscript and:

- Added the following paragraph to the start of the Results section: "We assessed the false negative rate by comparison to an established, exhaustive search strategy represented by Fit3D [Kaiser, 2015], a method based on rigid alignments that scores hits by R.M.S.D. values. We filtered the Fit3D result list for R.M.S.D. values <1 Å to identify true positive hits that our method should report in any case (these hits may not be biologically functional, but should be regarded as promising candidates meriting closer inspection). Our method also finds additional hits because the Fit3D web server operates on a redundancy filtered version of the PDB archive."
- Extended the last subsection of the Methods section by a more detailed description of what "false negatives" means in the context of our manuscript and also addressed the question about the "false positive rate" by the following addition: "[...] overall false negative rate (given as the number of hits reported by Fit3D but missing in result set of our method, divided by the total number of Fit3D hits below the 1 Å R.M.S.D. threshold). We do not discuss false positives (*i.e.*, hits found by our method but not by Fit3D) as they are merely the result of the mandatory redundancy filtering by Fit3D or recent additions to the PDB archive."

Remark 3: *I feel very puzzled by the description that the Authors choose for all the residue pairs. They report the identity of the two residues (i.e. DS for aspartic acid and serine) and then 3 integer numbers associated to the backbone distance (Calpha for amino acids), the side-chain distance (Cbeta for amino acids) and an angle defined by the two vectors connecting backbone and side-chain of these two residues. I do not like the choice of Cbeta as representative of any side-chain (from Trp to glycine), and I am not sure that the descriptor of the residue pair is symmetric with respect to the same pair positioned in a different order in the sequence, even if in the same relative position in space. I think that if this is true, this method would be unable to identify identities of 3D motifs in non-homologous proteins. Or also 3D motifs with residues in different chains, situated in reverse order in the PDB file.*

Response: We made the early design decision to support so-called position-specific exchanges that allow a set of amino acids at a defined position of the query motif (rather than only the exact amino acid observed in the query). The manuscript gives an example by the motif of the enolase superfamily. From an implementation perspective, we assumed all 20 amino acids to be valid substitutions for one another. A representation based on alpha and beta carbons is applicable for all amino acids (for glycine, the ideal beta carbon coordinates of alanine are used). This decision is a trade-off and, *e.g.*, emphasizes the position of alpha carbon and underrepresents the position of side-chain atoms, especially for larger amino acids.

Methods such as ASSAM (https://doi.org/10.1093/nar/gks401) successfully applied amino acid-specific representations that capture the position of side-chain atoms better. We investigated other representation schemes (*e.g.*, beta carbon paired with the heavy atom farthest away from any backbone atom) but observed a diminished ability to handle position-specific exchanges. This ultimately led us to the discussed descriptors based on alpha and beta carbons.

The presented geometric descriptors are symmetric: The computed angle values are order-independent and combinations of residue types are unordered (both an alanine followed by a cysteine and a cysteine followed by an alanine will be represented as 'AC'). Thank you for pointing out that the manuscript did not explicitly mention the symmetric quality of the geometric descriptors.

We made the manuscript more precise by:

- Stating that descriptors are symmetric in the first paragraph of the Results and discussion section
- Extending the "Geometric descriptor" subsection in the Method section by: "The presented geometric descriptors are symmetric, as in, independent from the sequence in which both residues appear. For increased storage efficiency, residue type information of descriptors is sorted lexicographically (any residue pair of an alanine and a cysteine is represented by 'AC', there is no bin 'CA')."
- Consolidating the scoring subsection of the Methods section by including: "An all-atom alignment can put too much emphasis on backbone atoms when chain directions differ, or a certain functionality is exclusively realized by sidechain atoms. In such cases, it may be beneficial to align only sidechain atoms or the atoms used to define the geometric descriptors."

Remark 4: *A minor weakness of this method is that it works with residue identities, while biologically meaningful 3D motifs usually also allow similar residues in the same positions.*

Response: We improved the manuscript so that it communicates more clearly that the definition of position-specific exchanges allows to define queries with sets of similar residues at a certain position. As outlined above, this was also our main motivation to represent amino acids generically by alpha and beta carbons.

We updated the manuscript by the following changes:

- Extended the first sentence on position-specific exchanges by some alternative wording: "Queries with position-specific exchanges allow a set of similar amino acids at the same position (as shown for the enolase superfamily template). Such queries require [...]"

Reviewer #3:

Remark 1: *In all presented test cases, authors have chosen a specific configuration for each query taken from specific PDB structures. I assume these configurations are the most common for each motif examined. However, I believe that analyzing how the choice the query configuration influences results would definitely add to the paper. The analysis could be also restricted to a single motif e.g. His-Asp-Ser motif.*

Response: Thank you for this interesting remark. Most motif definitions were taken from literature; however, examples such as the His2/Cys2 zinc fingers show that variations of the motif definition can change the performance and usefulness of our method.

We followed your proposal and analyzed how the reference structure (from which the query motif was extracted) influences the result set of our method. We have done this by taking the result set of the Zinc Finger motif and used each individual hit as a query definition of an independent search run. This resulted in 1,062 queries that were processed in less than 2 minutes.

We added these findings to the Result subsection on the Zinc Finger motif by:

- “To underscore this point, we investigated whether the simplified 3-residue search query can be refined further. The low runtimes of our method allow optimization of query definitions by using all accepted hits of an initial run as query definitions for individual, subsequent runs. Some query results will return fewer hits than the initial query, while others may report more or possibly different hits. For the zinc finger motif, more than 1,000 queries were processed within 111 s. The query based on PDB ID 2emb (Cys:A-15, His:A-31, His:A-35) returned the most hits and more than doubled the size of the result set to 2,261. PDB ID 5yef (Cys:D-36, His:D-49, His:D-54) features the largest addition of 1,571 previously unidentified hits, but also misses 676 hits that were captured by the initial query motif. PDB ID 5c8t (Cys:D-280, His:D-258, His:D-265) returns the smallest result set with only 208 hits. All of these motifs feature a coordinated zinc ion in the PDB structure. This experience demonstrates the importance of the query definition. In cases where exact geometry is subordinate, it may be beneficial to search for multiple query definitions and merge these results to produce a comprehensive, non-redundant set of PDB structures containing the structural motif in question.”

Remark 2: *In Tables 1 and S1 authors report search results for the five case studies. I suggest to also include results obtained with other approaches e.g. using the Fit3D method. This would provide the reader with a side-by-side comparison that would better highlight the advantages (in terms of time, hits and FNR) of the proposed approach over exhaustive search strategies.*

Response: We initially chose to omit runtimes of the Fit3D web server because it is difficult to provide an objective comparison (*e.g.*, because Fit3D operates on redundancy filtered subsets of the PDB archive and the hardware of the Fit3D server is unknown). However, it may very well be helpful to users to have access to the number of hits reported by Fit3D and expected processing times.

We updated the manuscript as follows:

- Added Fit3D hit counts and processing times as supplemental table 2
- Added a reference to S2_Table to the “runtime analysis” subsection: “As a comparison to an existing method, [S2_Table] summarizes the number of results and the required runtime by the Fit3D web server [Kaiser, 2016].”

Regarding false negative rates: Fit3D is an exhaustive method that will report all relevant hits that are similar to the query motif. Similarity is determined by a rigid all-atom alignment of query motif and candidate hit. We consider the hit as meaningful if the alignment exhibits a R.M.S.D. below threshold of 1 Å. We considered this set relevant and free of false negative hits (in the sense that all reported hits are valid alignments below the R.M.S.D. threshold, though they are not necessarily functional).
